# Relative levels of the proprotein and cleavage‐activated form of circulating human anti‐Müllerian hormone are sexually dimorphic and variable during the life cycle

**DOI:** 10.14814/phy2.12783

**Published:** 2016-05-04

**Authors:** Michael W. Pankhurst, Yih Harng Chong, Ian S. McLennan

**Affiliations:** ^1^ Department of AnatomyOtago School of Medical SciencesUniversity of OtagoDunedinNew Zealand; ^2^ Department of MedicineDunedin School of MedicineUniversity of OtagoDunedinNew Zealand; ^3^ Brain Health Research CentreUniversity of OtagoDunedinNew Zealand

**Keywords:** Age, bioactivity, proAMH, prohormone, serum, testes

## Abstract

Anti‐Müllerian hormone (AMH) is a gonadal hormone, which induces aspects of the male phenotype, and influences ovarian follicular recruitment. AMH is synthesized as a proprotein (proAMH), which is incompletely cleaved to the receptor‐competent AMH_N__,C_. AMH ELISAs have not distinguished between proAMH and AMH_N__,C_; consequently, the physiological ranges of circulating proAMH and AMH_N__,C_ are unknown. A novel proAMH ELISA has been used to assay serum proAMH in humans. Total AMH was also measured, enabling the AMH_N__,C_ concentration to be calculated. Stored serum from 131 boys, 80 younger, and 106 older men were examined, with serum from 14 girls and 18 women included for comparison. The mean levels of proAMH and AMH_N__,C_ in pM were respectively: boys (253, 526), men (7.7, 36), elderly men (5.7, 19), girls (3.3, 15), and women (5.2, 27) (boys vs. men, *P* < 0.001; girls vs. women, *P* = 0.032). The proportion of proAMH as a percentage of total AMH (API) was approximately twofold higher in boys than men (*P* < 0.001) with little overlap between the ranges, with girls also exhibiting lesser cleavage of their AMH than women (*P* < 0.001). The API varied within each population group. In young men, the API did not correlate with circulating levels of the other testicular hormones (testosterone, InhB, and INSL3). In conclusion, the cleavage of circulating AMH varies extensively within the human population, with most individuals having significant levels of proAMH. The physiological and clinical relevance of circulating proAMH needs to be established.

## Introduction

Anti‐Müllerian hormone (AMH) is a regulator of sexual differentiation and gonadal function. In males, it triggers the regression of the paramesonephric (Müllerian) duct (Josso 1992; MacLaughlin and Donahoe 2004), moderates aspects of testicular development (Behringer et al. 1990; Mishina et al. 1996), and contributes to the generation of the male phenotype in the lung (Catlin et al. 1997) and nervous system (Wang et al. 2009; Wittmann and McLennan 2013a,b). During human development, it putatively slows the speed of male development (Morgan et al. 2011) and modulates the severity of symptoms of boys with an autism spectrum disorder (Pankhurst and McLennan 2012). In females, the granulosa cells of developing, nonatretic ovarian follicles secrete AMH which regulates the depletion of the ovarian reserve and the sensitivity of developing follicles to FSH (Durlinger et al. 1999, 2001; Visser et al. 2007). The roles of gonadal hormones in adults extend beyond reproduction, to include the regulation and maintenance of nonreproductive tissues. AMH has rarely been considered in this context, but recent studies have linked circulating AMH levels with cardiovascular conditions in men (Dennis et al. 2012), and with the susceptibility to experimentally induced atherosclerosis in rhesus monkey females (Appt et al. 2012).

Anti‐Müllerian hormone is a member of the TGF*β* superfamily, which produces context‐dependent regulation through the interaction of multiple ligands with shared receptors and binding proteins (Shi and Massague 2003; Massague 2012). The TGF*β*s, like most other protein signaling ligands, are synthesized as proproteins that are cleaved to produce the receptor‐competent form. In case of AMH, the cleavage of the proprotein within the gonads is incomplete, with the result that circulating AMH is a mixture of proAMH and AMH_N,C_ (Pankhurst and McLennan 2013), with only the latter being able to activate the AMH‐specific receptor (AMHR2) (MacLaughlin et al. 1992; di Clemente et al. 2010). When recombinant‐proAMH is injected intravenously into mice, no AMH_N,C_ accumulates in the circulation, with the rate of clearance of the proAMH and AMH_N,C_ being similar (Pankhurst et al. 2016). Hence, the extent of the cleavage of circulating AMH appears to be determined by the gonads. The controlled cleavage of proproteins serve multiple functions, which may provide clues to the function of proAMH. For example, the bioactivity of TGF*β*1 can be suppressed by the targeted degradation of the proTGF*β*1 (Shiga et al. 2011). By analogy, circulating proAMH may be the product of a mechanism to suppress AMH bioactivity. On the other hand, proAMH appears to be cleaved by the Müllerian duct in vitro (Cate et al. 1986; Wilson et al. 1993), raising the possibility that the AMH‐receptor bioactivity of circulating AMH varies depending on the ability of target tissues to cleave proAMH (McLennan and Pankhurst 2015). If so, the concentration of circulating AMH_N,C_ represents the basal level of AMH bioactivity, whereas circulating proAMH represents the pool of context‐dependent bioactivity. Equally, some proproteins have bioactivity that is independent of their subsequent cleavage, with the proneutrophins being a well‐characterized example of this (Nykjaer et al. 2004; Hempstead 2014). Consequently, it is currently unclear whether proAMH and AMH_N,C_ are two distinct hormones, or two interrelated forms of a hormone whose bioactivity is partially determined by peripheral tissues.

The total level of AMH species (proAMH and AMH_N,C_) varies with sex, age, and the state of the gonads (Lee et al. 1996; Aksglaede et al. 2010; Hagen et al. 2010; Seifer et al. 2011). ProAMH is putatively cleaved in vivo by subtilisin/kexin‐type proprotein convertases (PCSK3 and 5) and plasmin (Pepinsky et al. 1988; Nachtigal and Ingraham 1996). The levels of these enzymes within the gonads are subject to complex regulation, which has sex‐specific and age‐related aspects (reviewed [McLennan et al. 2015]). This raises the possibility that the bioactivity of circulating AMH varies between individuals, due to regulated differences in the extent to which proAMH is cleaved before release into the circulation. We have developed a proAMH‐specific ELISA (Pankhurst and McLennan 2016) to investigate this possibility, and report that the relative proportion of proAMH to total AMH varies during the life cycle and between the sexes, with lesser variation occurring between individuals of a similar age and sex. In young men, the extent of cleavage was unrelated to the levels of the other testicular hormones.

## Material and Methods

### Participants

The samples from this study were stored samples, obtained from prior studies (Morgan et al. 2011; Chong et al. 2013). The serum when collected was snap frozen in single‐use aliquots in liquid nitrogen and stored at −80°C, for up to 3 years. Freeze–thawing of samples does not affect the AMH ELISA (Kumar et al. 2010).

Both children and adult participants were separately recruited from the community in Dunedin, New Zealand, via flyers and newspaper advertisements. The five study groups: girls, boys, women, young men, and older men were defined based on sex and age. Age characteristics for each group are provided in Table 1. All participants were community‐dwelling with no prior history of endocrine or reproductive disorders. This project was approved by the University of Otago Human Ethics Committee (Health) and was conducted in accordance with the principles set out in the Declaration of Helsinki. Written informed consent was obtained from each adult participant and the legal guardian of each child participant. In the adult female cohort, donors were requested to donate blood within 2 weeks of the onset of menstrual bleeding. The time since menstruation was not recorded, and the stage of the ovarian cycle was not verified by hormone analysis.

**Table 1:** Study sample age characteristics

	Boys	Men < 50 years	Men > 50 years	Girls	Women
*n*	131	80	106	14	18
Mean (SD)	6.7 (1.1)	40.2 (6.5)	73.6 (9.1)	7.5 (1.5)	31.7 (8.5)
Range	4.5–10.5	24.5–49.3	50.5–89.7	4.4–10.1	19.0–45.9

The data are expressed in years.

Total AMH was measured in duplicate using the AMH Gen II assay (Beckman Coulter, Cat# A79765, following field safety notice FSN‐20434‐3, June 2013) according to the manufacturer's specified protocol. ProAMH was measured according to a modified protocol of the Gen II assay, described previously (Pankhurst and McLennan 2016). The AMH Gen II calibrators (Beckman Coulter, Cat# A79766) were used for quantification in total AMH immunoassays and a recombinant human proAMH standard was used for quantification in proAMH immunoassays (Pankhurst and McLennan 2016). A recombinant human AMH_N,C_ negative control (Pankhurst and McLennan 2016) was included in the ELISA run. The intra‐assay % coefficient of variations were 5.4 and 5.2 for the total AMH and proAMH ELISAs, respectively. The levels of Inhibin B (InhB), insulin‐like peptide 3 (INSL3) and testosterone were quantified as recently described (Chong et al. 2015). Commercially available ELISAs were used to assay InhB (Beckman Coulter, A81303) and INSL3 (Phoenix Pharmaceuticals, FEK‐035‐27). Testosterone levels were measured by the Canterbury Health Laboratory using an in‐house ELISA method (Elder and Lewis 1985). ELISA sample concentrations were calculated from standard curves fitting to quadratic equations (Prism 6, Graphpad Software).

### Data analysis

The AMH prohormone index (API) was calculated as the relative proportion of proAMH, expressed as a percentage of total AMH (API = [proAMH]/[total AMH] × 100). The level of AMH_N,C_ was estimated as [total AMH] − [proAMH]. Comparisons of the mean levels of total AMH concentrations, proAMH concentrations, and API were conducted using a generalized linear model with Bonferroni post hoc tests, when multiple groups were compared. Student's *t*‐tests with equal variance assumed when Levene's test indicated homoscedasticity of data were used when two groups were compared, with an a priori hypothesis. Correlations were carried out using the Pearson method. The association between testicular hormones and the API was examined using Pearson linear regression. All analyses were conducted using SPSS v22.0 (IBM).

## Results

### ProAMH levels in males

**Figure 1:**
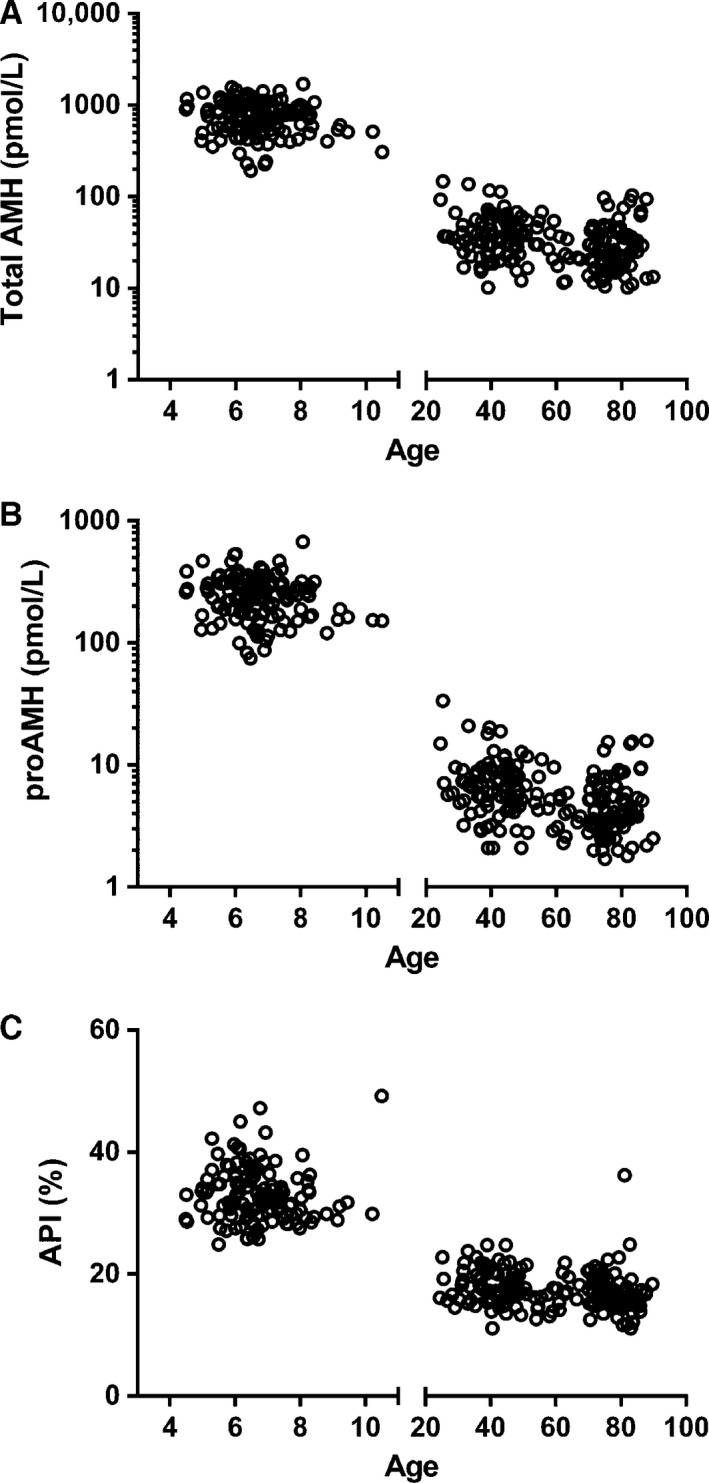
Total AMH (A), proAMH (B), and API (C) in boys aged 4–11 (*n* = 131) and men aged 24–90 years (*n* = 186). Note, the log‐scale of the *y*‐axis in (A) and (B). Significant differences between boys and men were detected for total AMH (*P* < 0.001), proAMH (*P* < 0.001), and API (*P* < 0.001). API is a measure of the relative proportion of uncleaved AMH as defined in the Methods. 1 ng/mL AMH = 7.14 pmol/L (pmole/L).

The total AMH levels (Table 2) were consistent with previous studies (Lee et al. 1996; Aksglaede et al. 2010). Boys had significantly higher levels of both proAMH (*P* < 0.001) and AMH_N,C_ (*P* < 0.001) than men (Fig. 1, Table 2). Total AMH levels exhibited an age‐related decline in older men (Chong et al. 2013). There were also negative correlations between age and the variables proAMH (*r* = −0.313, *P* < 0.001) and AMH_N,C_ (*r* = −0.223, *P* = 0.002) indicating that both forms of AMH are less abundant in older men. The adult men were stratified into young (24–50 years) and older (>50 years) groups for subsequent analysis to examine age‐related differences in proAMH relative to AMH_N,C_.

**Table 2:** Levels of proAMH, total AMH, and calculated AMH_N,C_

	Boys	Men < 50 years	Men > 50 years	Girls	Women
*n*	131	80	106	14	18
Total AMH (pmol/L)					
Mean (SD)	779.1 (306.0)^a^	43.2 (26.2)	31.8 (19.8)	24.4 (14.9)	17.9 (8.0)
Range	192.5–1708	10.2–147.2	4.3–103.5	6.7–57.3	4.9–50.4
proAMH (pmol/L)					
Mean (SD)	253.1 (100.2)^a^	7.7 (4.9)	5.2 (3.1)	5.7 (3.4)	3.3 (1.7)
Range	74.9–674.4	2.1–33.5	4.3–103.5	1.6–13.4	0.8–9.1
AMH_N,C_ (pmol/L)					
Mean (SD)	526.0 (215.1)^a^	35.5 (21.7)	26.6 (16.9)	18.7 (11.5)	14.6 (6.4)
Range	117.7–1106	8.1–116.4	1.3–15.8	5.1–43.9	4.1–41.3
API (%)					
Mean (SD)	33.0 (4.4)^a^	18.1 (2.7)	17.0 (3.4)	23.7 (2.9)^a^	17.7 (2.6)
Range	24.9–49.2	11.2–24.9	11.1–36.4	17.9–28.3	13.3–22.4
proAMH/AMH_N,C_					
Mean (SD)	0.49 (0.11)^a^	0.22 (0.04)	0.21 (0.06)	0.31 (0.05)^b^	0.22 (0.04)
Range	0.33–0.97	0.13–0.33	0.12–0.57	0.22–0.40	0.15–0.29

1 ng/mL AMH = 7.14 pmol/L (pmole/L). The groups were compared using a generalized linear model, with Bonferroni post hoc tests.

a Significantly different to all other groups, *P* < 0.001.

b Significantly different to each of the male groups *P* < 0.001, and *P* = 0.005 to the other female groups.

The mean AMH prohormone index (API, see Methods) represents the relative proportion of proAMH expressed as a percentage of total AMH. The mean API was almost twofold higher in boys (33.0 ± 4.4) than in young men (18.1 ± 2.8), with this difference being highly significant (*P* < 0.001, Table 2, Fig. 1). The API was also significantly lower in older men relative to boys (*P* < 0.001) and slightly lower than younger men (*P* = 0.017). These data indicate that the total AMH in men is comprised of a higher proportion of AMH_N,C_ than the total AMH in boys. The differences in API between boys and young men were so distinct that there was no overlap in the ranges of the API in young men (11.1–24.8) and boys (24.9–49.2). The distribution of API in older men was very similar to young men with the exception of an 81‐year‐old individual who displayed a boy‐like API of 36.2.

### ProAMH levels in females

**Figure 2:**
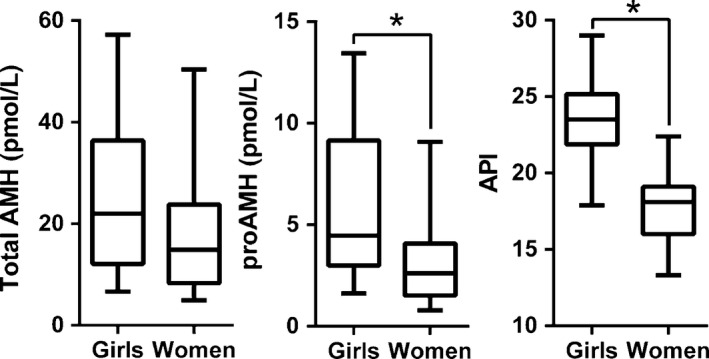
Medians, interquartile intervals, and ranges for total AMH, proAMH, and the API in girls aged 4–11 (*n* = 14) and women aged 19–46 (*n* = 18). Significant differences between girls and women were detected between proAMH levels (*P* = 0.032) and the API (*P* < 0.001). 1 ng/mL AMH = 7.14 pmol/L (pmole/L).

**Figure 3:**
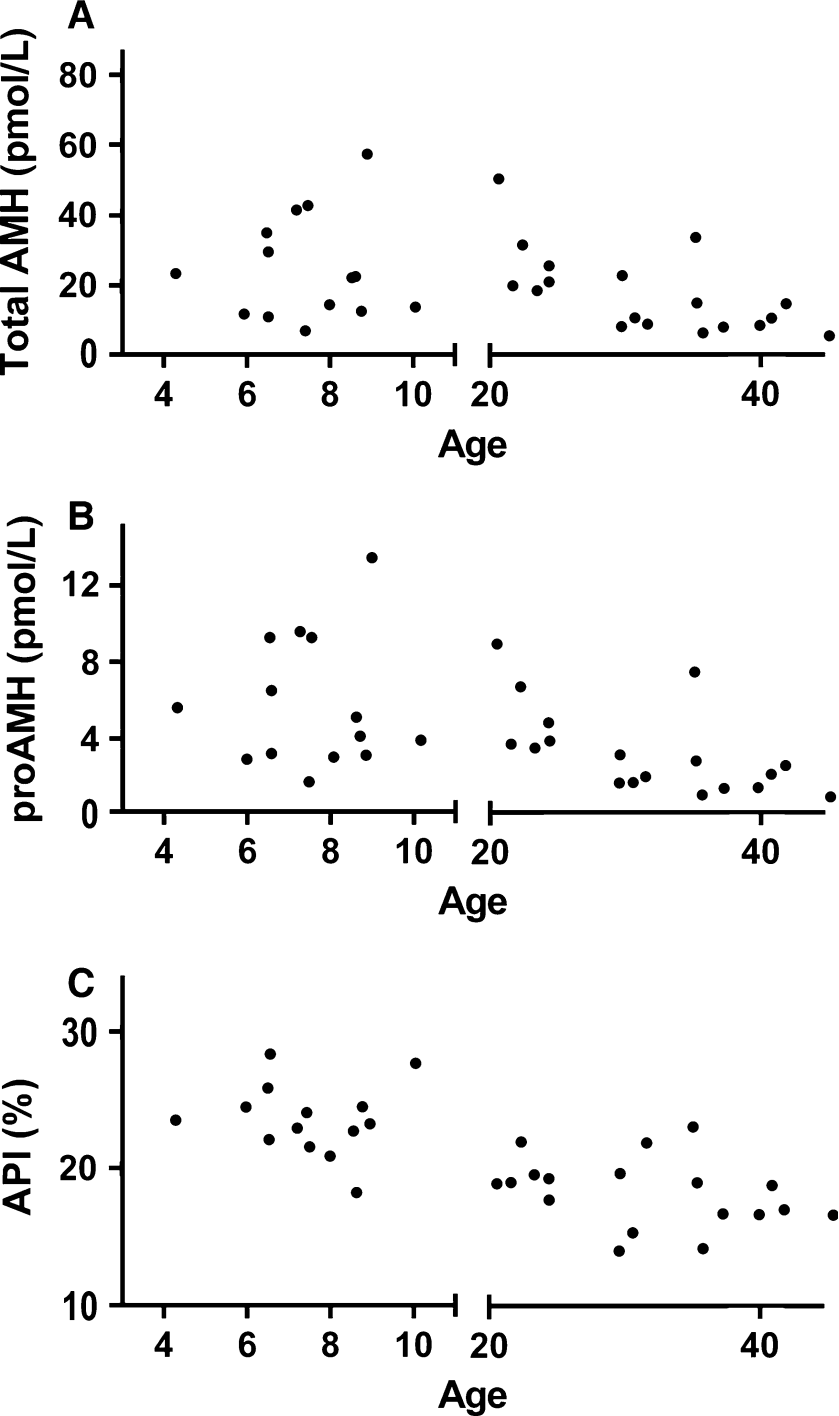
Total AMH (A), proAMH (B), and API (C) in girls and women aged. API is a measure of the relative proportion of uncleaved AMH as defined in the Methods. The API was significantly different between girls and women, *P* < 0.001. 1 ng/mL AMH = 7.14 pmol/L (pmole/L).

The circulating AMH in girls and women was also a mixture proAMH and AMH_N,C_, at all ages (Figs. 2, 3). Women, on average, had lower levels of both circulating proAMH and AMH_N,C_ than girls (Table 2), although this mainly reflects the marked decline in total AMH levels associated with depletion of the ovarian reserve (see [Seifer et al. 2011] and Fig. 3). The API and proAMH/AMH_N,C_ ratio in girls were significantly higher than in women. However, the magnitude of the difference between girls and women was less than the difference between boys and men, with the API and the proAMH/AMH_N,C_ ratio in boys being significantly higher than that of girls (Table 2). In contrast, there was no overt sexual dimorphism in the ratio of the two forms of circulating AMH in young adults, even though the absolute levels of AMH become increasingly dimorphic with age.

### The relationship between ProAMH and AMH_N,C_

**Figure 4:**
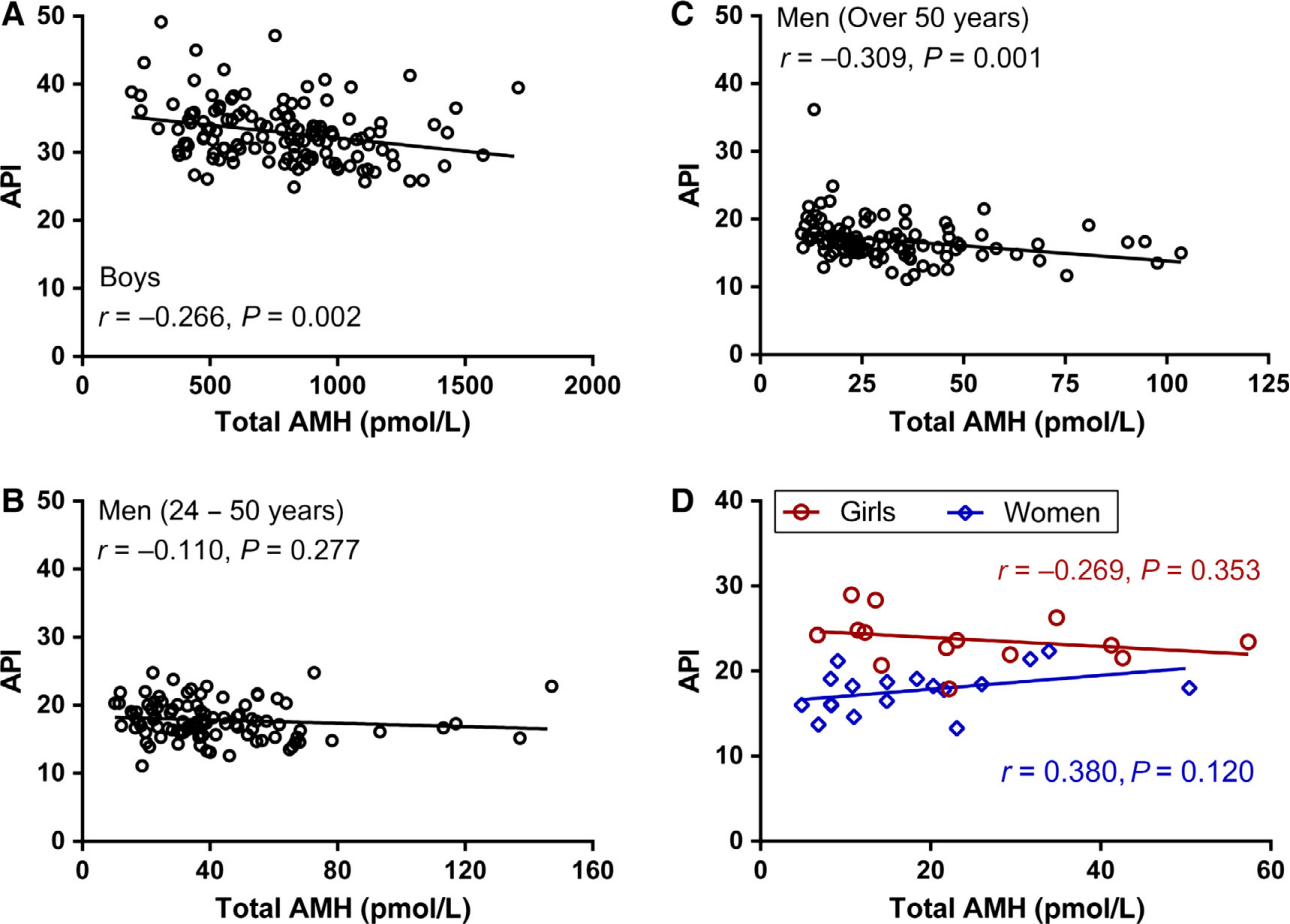
Correlates of total AMH concentrations with API in boys (A), men aged 24–50 years (B), men over 50 years (C) girls (D, red circles), and women (D, blue diamonds). Pearson's correlation coefficients (*r*) and *P*‐values are also displayed. 1 ng/mL AMH = 7.14 pmol/L (pmole/L).

**Figure 5:**
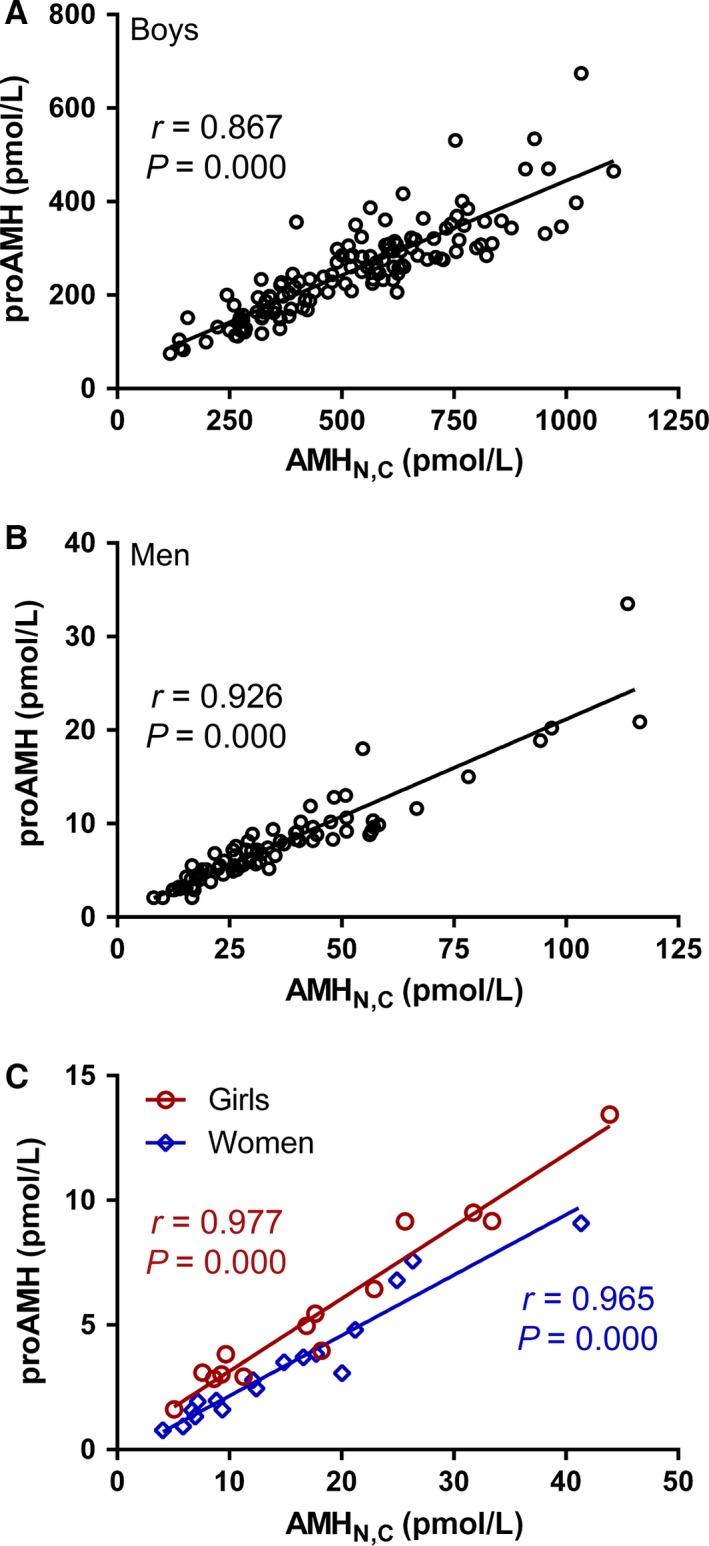
Correlates of proAMH with AMH_N__,C_ concentrations in boys (A), men (24–50 years) (B), girls (C, red circles), and women (C, blue diamonds). Pearson's coefficients (*r*) and *P*‐values are also displayed. 1 ng/mL AMH = 7.14 pmol/L (pmole/L).

There were significant, negative correlations between API and total AMH concentration in boys (*r* = −0.266, *P* = 0.002, Fig. 4A) and men older than 50 (*r* = −0.309, *P* = 0.001, Fig. 4C) indicating that the variation in total AMH levels explains only 7% and 10% of the variability in API, respectively. No correlation was observed between API and total AMH concentration in girls, women, or men younger than 50 years (Fig. 4B and D) indicating that the total quantity of AMH was independent of the relative proportions of cleaved AMH_N,C_ and uncleaved proAMH in these groups. Accordingly, proAMH and AMH_N,C_ concentrations were highly correlated in girls (*r* = 0.977, *P* < 0.001, Fig. 5), boys (*r* = 0.867, *P* < 0.001, Fig. 5), young men (*r* = 0.926, *P* < 0.001, Fig. 5), and women (*r* = 0.965, *P* < 0.001, Fig. 5), despite variation being present within each population group.

### The API does not associate with the levels of testicular hormones in men

The testes of boys and men differ in their levels of testicular hormones, with boys having only trace levels of Leydig cell hormones (androgens and INSL3) (Wudy et al. 1995; Ivell et al. 2014), and low levels of inhibin B (InhB) (Raivio and Dunkel 2002) compared to men. Each of these testicular hormones has actions within the testes (Hedger and Winnall 2012; Ivell et al. 2014; Smith and Walker 2014), raising the possibility that one or more of them up‐regulates the cleavage of AMH, leading to a lower API in adults. If so, the API of adult men should negatively correlate with the level of their testicular hormones. In young men, the API was not significantly associated with testosterone, INSL3, or InhB (Table 3).

**Table 3:** The relationship between API and circulating levels of other testicular hormones in young men

Hormone	*R*	*P*
Testosterone	0.066	0.57
INSL3	0.040	0.73
Inhibin B	0.116	0.30

The association between each of the testicular hormones and the API was examined by Pearson linear regression, with the *R* and *P* values recorded.

## Discussion

The proportions of proAMH and AMH_N,C_ in the circulation vary within the population. This is most evident in the difference in API between boys and men, but a significant difference was also observed between girls and women. This indicates that a greater proportion of the total AMH in the blood of adults consists of the receptor‐competent AMH_N,C_. The API also varies between similarly aged individuals of the same sex.

### Abundance of circulating proAMH

AMH_N,C_ was more abundant in the circulation than proAMH in all individuals examined. However, the proportion of circulating AMH that is in the precursor form (proAMH) is qualitatively different to other hormones. The fasting proinsulin:insulin ratio, for example, is typically 0.03–0.07, and rarely rises above 0.10 (Bryhni et al. 2010; Guettier et al. 2013). In contrast, the mean proAMH:AMH_N,C_ ratio was as high as 0.25 in healthy young adults, and exceeded 0.30 in most boys and some older men. In absolute terms, the concentration of proAMH ranged from 1 pmol/L to 0.67 nmol/L in the population. This is within the plausible signaling range for a TGF*β* superfamily ligand, as signaling of this family is context‐dependent (Massague 2012), with some ligands exhibiting multiple dose–response curves, enabling signaling to occur at concentrations that span sub pmol/L to nmol/L (Roberts and Sporn 1990). The significance of the high abundance of proAMH is currently unproven, but points to the possibility that proAMH is either a functional regulator in its own right and/or is converted to AMH_N,C_ in extragonadal tissue.

### Circulating AMH in children

Boys have high levels of total AMH, but have less complete cleavage of proAMH. This could arise if the production of proAMH in boys exceeded the capacity of the gonads to cleave it. However, there was a statistically significant trend for the boys with the highest levels of total AMH to exhibit more complete cleavage of proAMH. Furthermore, girls and women have a similar range of total AMH levels, but differ in the extent to which they cleave their AMH. These observations indicate that the extent to which proAMH is cleaved is partially or totally independent of the factors that control how much AMH is produced. The consequence of this is that the dichotomy between boys on one hand, and men, girls, and women on the other, is greater for AMH_N,C_ than for proAMH, although there is a boy‐bias in the abundance of both forms of AMH.

### Testicular regulation of proAMH cleavage

Intravenous injection of proAMH into mice does not lead to the accumulation of AMH_N,C_ in the circulation, suggesting that circulating AMH_N,C_ was cleaved prior to release from the gonads (Pankhurst et al. 2016). If so, the cleavage of proAMH may be enhanced by some process of gonadal maturation, which drives known changes in the expression of the enzymes that putatively cleave proAMH (reviewed [McLennan et al. 2015]). In males, the maturation of the testes involves changes in the germ cells, the state of the endocrine cells, and the profile of testicular hormones (down‐regulation of AMH and up‐regulation of testosterone, InhB, and INSL3). In young men, the levels of testosterone, InhB, and INSL3 did not associate with the API, suggesting that these hormones do not acutely regulate the extent of proAMH cleavage. Further confirmation of this is needed, as the critical measurement is the bioactivity of the hormone within the testes, which may differ from the circulating level of hormone measured by an ELISA. Similarly, there is a need for the influence of germ cells and the maturation state of Sertoli cells on the cleavage of proAMH to be investigated. The dichotomy in API between boys and men in this cross‐sectional study suggests that API changes during puberty. The rate of change in API is best profiled in a longitudinal design during the pubertal transition.

### AMH cleavage in older men

The majority of elderly men had API values within the range for younger men, despite having lower levels of AMH. In a small minority of elderly men, the API and proAMH: AMH_N,C_ ratio was higher, approaching boy‐level values for reasons that are undetermined. The magnitude of this effect was similar to the age‐associated increase in the fasting proinsulin:insulin ratio (Roder et al. 1998). The increase in the fasting proinsulin:insulin ratio occurs in pancreatic beta‐cell dysfunction (Roder et al. 1998; Pfutzner and Forst 2011), and is associated with insulin resistance, type 2 diabetes (Pfutzner et al. 2004; Loopstra‐Masters et al. 2011), and an increased risk of first stroke (Lindahl et al. 2000) or first acute myocardial infarction (Lindahl et al. 1999) (see also [Zethelius et al. 2002]). The enzymes which putatively cleave proAMH are not specific to AMH, with the consequence that the API may be also be a measure of the extent of cleavage of multiple proproteins. This points to the need for the influence of age‐related deterioration of Sertoli cells on their ability to process testicular proproteins to be studied.

### Limitations of AMH ELISAs

This study is an accurate representation of population differences, as the standards for the proAMH and total AMH assays exhibit parallel dose–response characteristics (Pankhurst and McLennan 2016). However, there is currently no international AMH standard that can be used to calibrate and harmonize measurements. Therefore, comparisons of absolute AMH values cannot currently be made between different total AMH assay platforms. International standards for total AMH are currently being considered by the World Health Organization. To avoid the same issue with proAMH or AMH_N,C_ assays, we advocate for the generation of separate standards of pure proAMH and pure AMH_N,C_. In the absence of reference standards, local reference ranges will be needed for investigations of proAMH, AMH_N,C_, and the API. The relationships observed between population groups should, however, be replicable between studies using different assay platforms.

### Interpretation of correlations between AMH species

The total AMH and AMH_N,C_ levels in young and perimenopausal women are highly correlated, leading to the argument that the processing of AMH may be relatively invariant between individuals (Robertson et al. 2014). However, the extent to which AMH_N,C_ or proAMH correlate to total AMH levels is an insensitive method for detecting variation in AMH processing. The interperson variation in circulating AMH concentration is large, whereas the proportion of AMH_N,C_ and proAMH that comprise total AMH is confined to a narrow range, that is, a fraction, mathematically constrained between 0 and 1 (i.e., 0–100% in the case of API). This will generate strong correlations between AMH_N,C_ and total AMH that are primarily due to the large concentration variability, with variation in the forms of AMH having little impact. Consequently, future studies of AMH processing during the pubertal transition (both sexes), the ovarian cycle, pregnancy, lactation, and the menopausal transition will need to be longitudinal, to enable any stage‐specific events to be quantified in the presence of the large person‐to‐person variation in absolute levels of the AMH species.

In this study, the extent of cleavage of AMH (API) did not correlate with total AMH levels in females or young men, and with total AMH levels in boys explaining less than 10% of the biological variation in API. This indicates that the factors which control the cleavage of proAMH are largely or totally distinct from the factors that determine the level of AMH. Hence, any measurement of the extent of cleavage needs to be independent of AMH level. This situation is analogous to the use of proinsulin as a biomarker of pancreatic beta‐cell function, where the proinsulin:total insulin ratio or the proinsulin:insulin C peptide ratio have proven utility.

## Conclusion

Significant levels of proAMH are present in the circulation with a pattern that varies between the sexes, with the stage of the life cycle, and between similar individuals. This study defines the physiological range for circulating levels and lays a foundation for future studies into the function(s) of proAMH and the mechanisms that regulate its levels.

## Conflict of Interest

ISM and MWP have filed a patent pertaining to cleavage‐state‐specific AMH assays including the proAMH‐specific assay used in this manuscript (WO 2014/204327 A1).
